# Anterior Mandibular Segmental Distraction Osteogenesis: A Case Report

**DOI:** 10.2174/1745017901814010623

**Published:** 2018-09-28

**Authors:** Thomas Starch-Jensen, Annette Dalgaard Kjellerup

**Affiliations:** Department of Oral and Maxillofacial Surgery, Aalborg University Hospital, Aalborg, Denmark

**Keywords:** Bone lengthening, Dentistry, Facial bones, Orthodontics, Orthognathic surgery, Class II malocclusion

## Abstract

**Introduction:**

Skeletal Angle Class I patients with a severe dental Class II malocclusion are characterized by an unfavourable anterior-posterior relationship between the anterior dentoalveolar area and the skeletal base. Orthodontic alignment posing various treatment difficulties and surgical correction with bilateral sagittal split osteotomy may result in a compromised facial profile. Hence, anterior mandibular segmental distraction osteogenesis has been proposed as an alternative treatment modality for solving facial esthetics, anterior tooth crowding and an unfavourable relationship between the anterior dentoalveolar area and the skeletal base in skeletal Angle Class I patients with a severe dental Class II malocclusion. Limited skeletal relapse with predictable soft tissue changes have been documented in long-term studies. Thus, anterior mandibular segmental distraction osteogenesis seems to be a valuable and predictable surgical method for correction of selected cases of skeletal Class I patients with a severe dental Class II malocclusion.

**Case report:**

The purpose of this case report is to present the treatment of a 57-year-old female with a skeletal Angle Class I relation and a severe dental Class II malocclusion. Anterior mandibular segmental distraction osteogenesis as well as discussing the current knowledge about this treatment modality.

**Conclusion:**

The present case report illustrates that establishment of a harmonious relationship between the maxillary and mandibular arch in patients with a skeletal Angle Class I relation and a severe dental Class II malocclusion using anterior mandibular segmental distraction osteogenesis seems to be a predictable and applicable surgical method for selected cases and General Dental Practitioners, orthodontics and maxillofacial surgeons must have knowledge of this treatment modality.

## 1 INTRODUCTION

Angle Class II malocclusion is the most prevalent sagittal skeletal discrepancy and commonly treated successfully with conventional orthodontic fixed functional appliance therapy, tooth extractions or orthognathic surgery [1]. However, orthodontic correction of skeletal Angle Class I patients with a severe dental Class II malocclusion posing various treatment difficulties including the risk of gingival recession, root fenestrations and loosening of teeth. Therefore, various surgical techniques for mandibular advancement have been advocated to overcome these periodontal and skeletal problems including bilateral sagittal split osteotomy, anterior segmental subapical osteotomy and Anterior Mandibular Segment Distraction Osteogenesis (AMSDO) [2-12]. Surgical correction of skeletal Angle Class I patients with a severe dental Class II malocclusion with a bilateral sagittal split osteotomy and advancement of the mandible may result in a compromised facial profile and anterior segmental subapical osteotomy only solves minor discrepancies between the dentoalveolar area and the skeletal base due to restricted soft tissue expansion. Hence, AMSDO of the alveolar process has been proposed as a valuable treatment option in selected cases: 1) Correction of skeletal Angle Class II patients with crowding to reduce the required sagittal distance to be achieved by an advancement bilateral sagittal split osteotomy, 2) Skeletal Angle Class III patients to create space for the decompensation of the lower incisor inclination, 3) Skeletal Angle Class I patients with a dental Class II malocclusion to create space of one premolar width and overjet normalization, and 4) Skeletal and dental Angle Class I patients with crowding to avoid extraction and the resulting unfavourable profile for correction of anterior tooth crowding and/or an unfavourable anteroposterior relationship between the anterior dentoalveolar bone and the skeletal base [3, 5, 9]. AMSDO was initially introduced by Triaca in 2001 [8], and limited skeletal relapse and predictable soft tissue changes have been documented in long-term studies [3, 5, 6].

The purpose of the present case report is to present another case of AMSDO and to summarize the current knowledge about this treatment modality.

## 2 CASE PRESENTATION

**Fig. (1):**
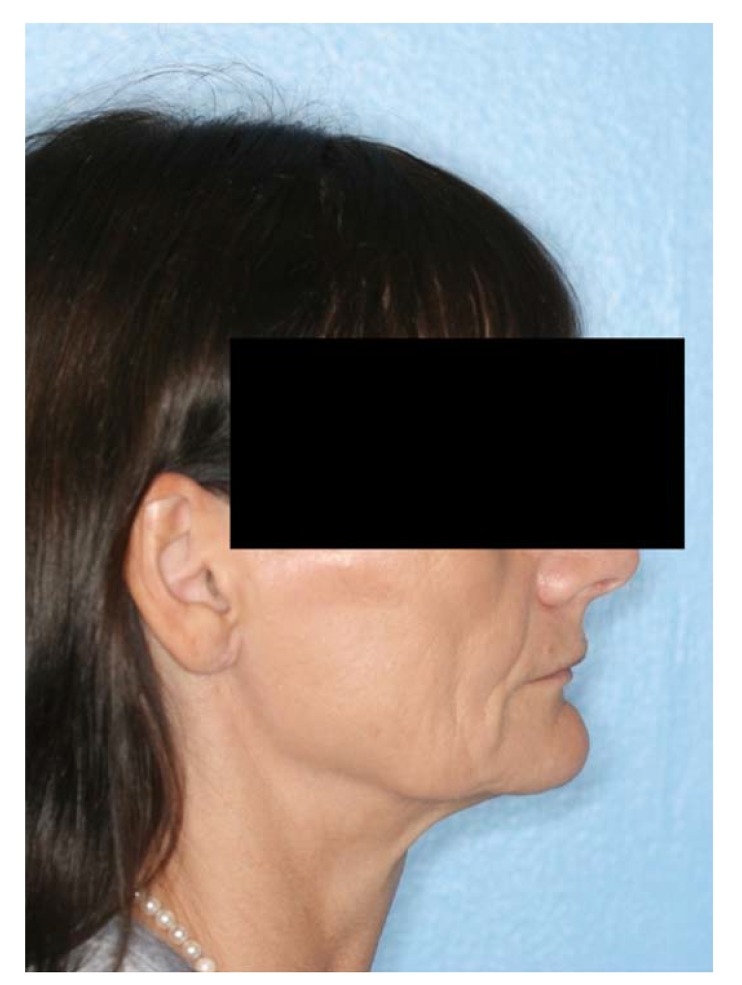
Clinical photo showing a decreased lower anterior facial height with the lower lip placed behind the Rickett's E-line and a prominent chin.

**Fig. (2):**
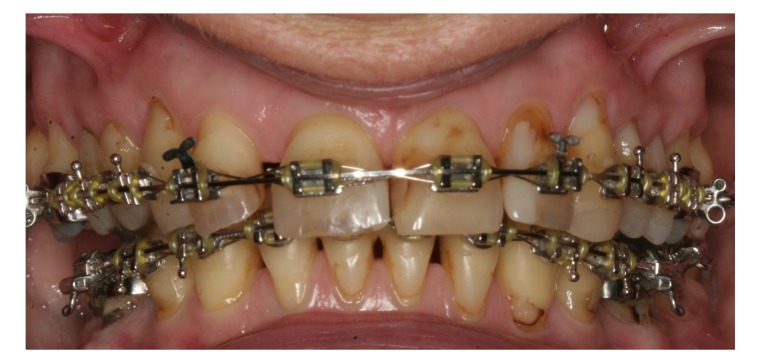
Intra-oral clinical photo showing an exaggerated curve of Spee.

**Fig. (3):**
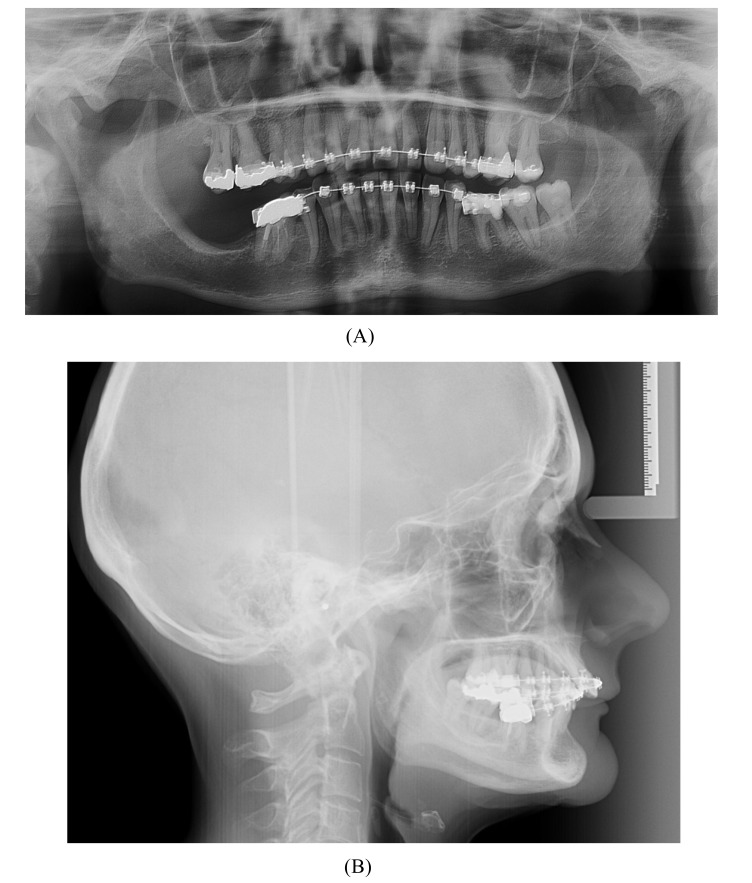
Radiologic examination. (**A**) Orthopantomogram showing an exaggerated curve of Spee. (**B**) Lateral cephalogram showing mandibular dental retrusion in combination with a deep bite.

A 57-year-old female complaining of masticatory and functional problems was referred by her orthodontist to the Department of Oral and Maxillofacial Surgery, Aalborg University Hospital, Denmark, for surgical correction of a skeletal Angle Class I relation with a dental Class II malocclusion due to the lack of orthodontic treatment effect. The patient had begun an orthodontic treatment a year ago. The patient´s medical history was unremarkable. Clinical examination demonstrated a lower anterior facial height with the lower lip placed behind the Rickett's E-line and a deep labiomental fold (Fig. **1**). Intra-oral examination revealed an exaggerated curve of Spee with an overjet and overbite of 8 mm and 7 mm, respectively. Minor buccal gingival recessions without probing pocket depths were observed around the mandibular incisors (Fig. **2**). Radiographically, orthopantomogram and lateral cephalogram revealed mandibular dental retrusion in combination with a deep bite and a prominent chin (Fig. **3**). The treatment approach plan including AMSDO to create space for tooth alignment and later placement of dental implants was presented to the patient and accepted.

**Fig. (4):**
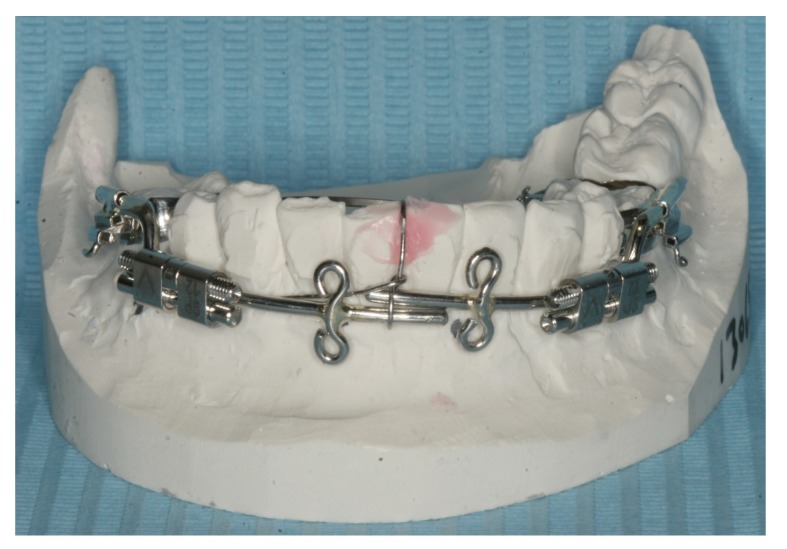
A custom-made, rigid, stainless steel, distraction device was fabricated.

Preoperative orthodontic treatment involved fixed orthodontic appliances to increase the inter-root space between the canines and first molars for the planned vertical osteotomies. A rigid custom-made distraction device was fabricated (Fig. **4**). The distraction device consisted of an anterior segment and the posterior distraction segment. The distraction device was fixed in tubes on to the buccal surfaces of the molar bands and the expansion screws were positioned parallel to the occlusal plane of the lower arch.

**Fig. (5):**
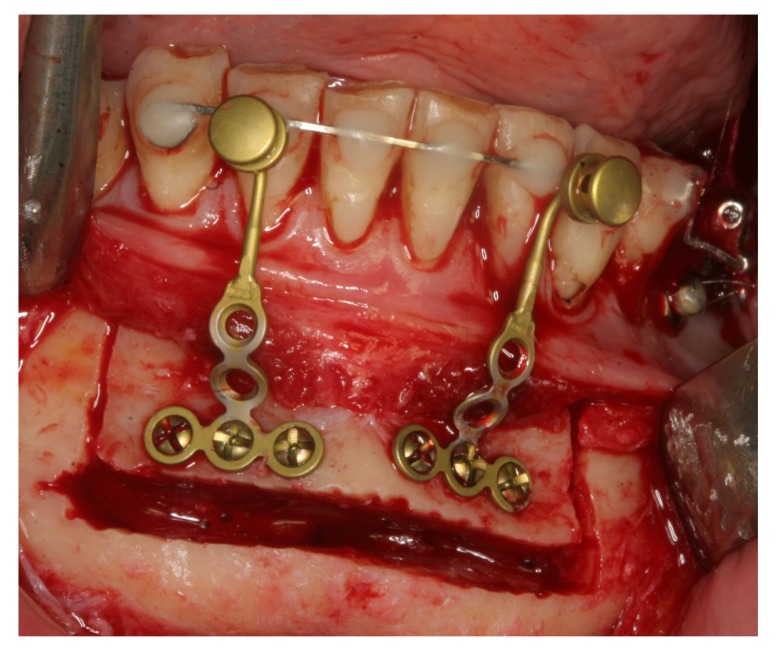
Intraoperative clinical photo illustrating the osteotomies and the bone anchors fixated to the tooth bearing segment.

**Fig. (6):**
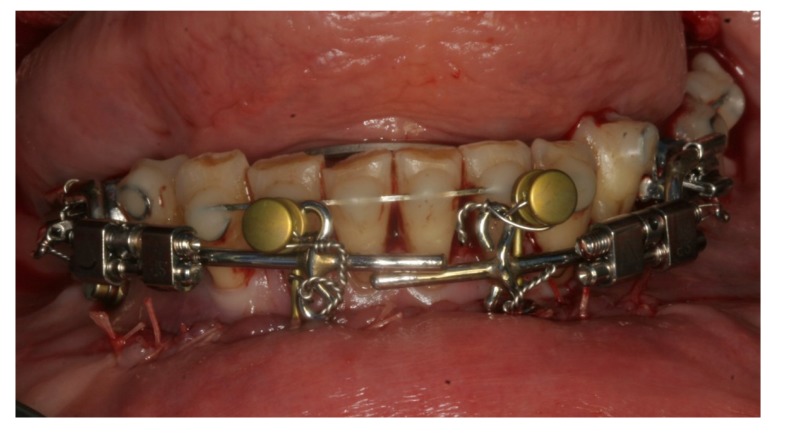
The bone anchors ligated to the distraction device.

**Fig. (7):**
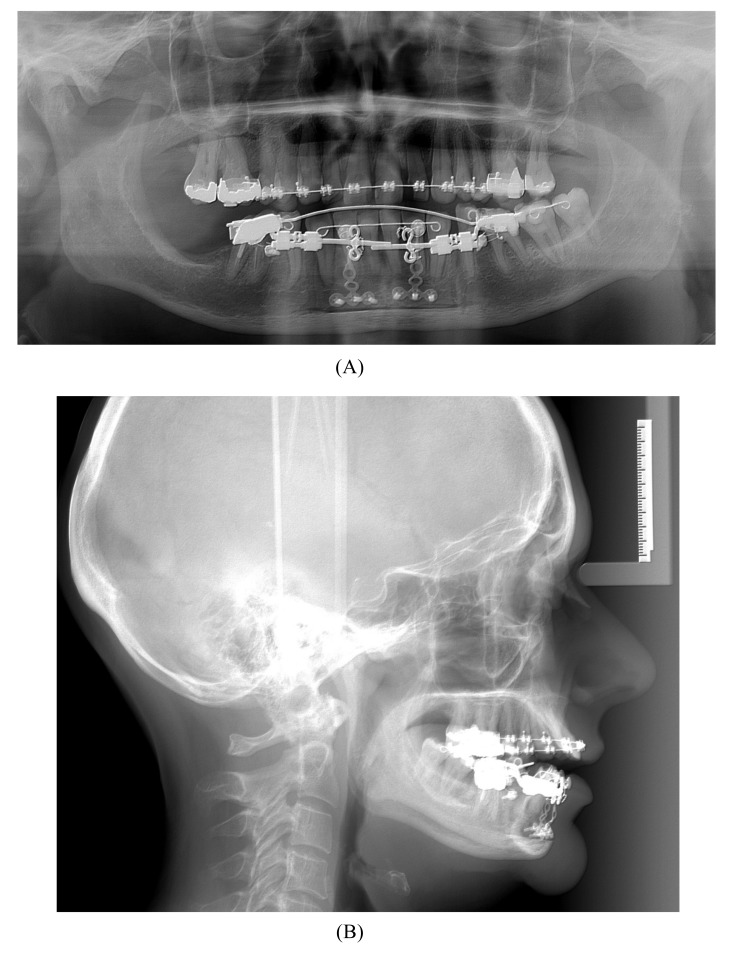
Postoperative X-rays. (**A**) Orthopantomogram showing satisfying osteotomy and position of the bone anchors. (**B**) Lateral cephalogram showing satisfying vector of the distraction device.

The surgical procedure was performed in general anaesthesia with nasotracheal intubation, supplemented by local anaesthesia. An intraoral vestibular incision was made from the right mandibular first premolar to the mandibular left first premolar. The mucoperiosteum was reflected, exposing the mandibular symphyses and mental foramens. Two horizontal osteotomies were made with piezoelectric surgery from the right canine to the left canine, at least 5 mm below the dental apices. Vertical osteotomies were made with piezoelectric surgery between the two horizontal osteotomies and a bone block of approximately 4.0 x 1.0 cm was removed with care to maintain the lingual periosteum and mucosa intact. Then, incomplete vertical osteotomies were performed with piezoelectric through the outer cortex between the canines and first premolars without detaching the dental papillae from the alveolar bone. The osteotomies were completed with a fine chisel until the anterior dentated segment could be mobilized without any bony resistance. The dentated anterior segment was repositioned in a more caudal position to level the dental arch. Two T-plate 9 mm bone anchors (Orthodontic Skeletal Anchorage System, Stryker Craniomaxillofacial, USA) were contoured to the outer cortex of the tooth-bearing segment and fixed with 5 mm monocortical screws (Fig. **5**). The bone anchors were ligated to the distraction device with wires and tested to ensure that the tooth-bearing segment was moving in a parallel direction without resistance (Fig. **6**). The distraction device was activated until a 1 mm diastema was achieved between the canines and the first premolars. The wound was irrigated with saline and the mucosa was re-adapted and sutured with resorbable sutures (Vicryl 3-0, Ethicon, Norderstedt, Germany). The patient was discharged later the same day. Postoperative x-rays disclosed satisfying osteotomies and placement of bone anchors (Fig. **7**). The postoperative period and healing was uneventful.

**Fig. (8):**
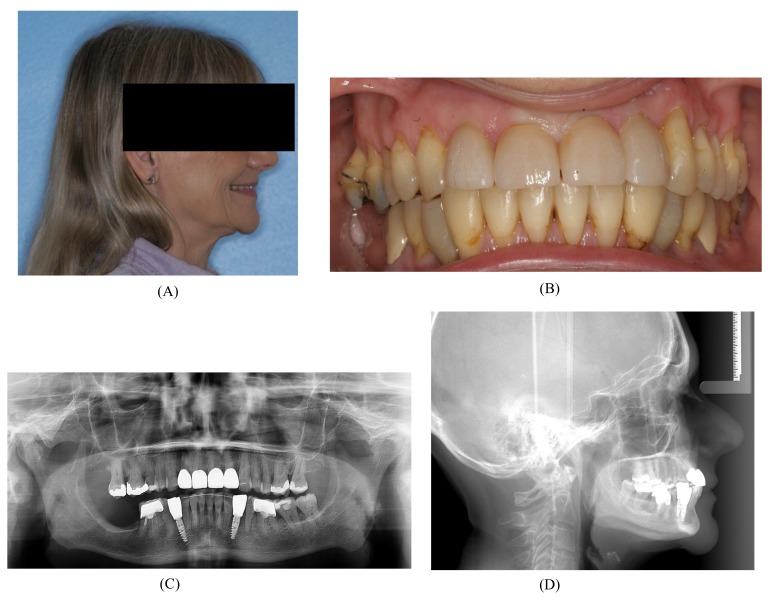
Final treatment result. (**A**) Clinical photo showing satisfying sagittal facial relations. (**B**) Intra-oral clinical photo showing the postoperative occlusion. (**C**) Orthopantomogram showing implant placement between the canines and first premolar. (**D**) Lateral cephalogram showing satisfying sagittal relations with normal incisor occlusion.

After a latent period of 5 to 7 days, the patient was instructed to activate the distraction device by 0.33 mm three times a day until the planned expansion was achieved. After the distraction phase was completed, a temporary composite tooth was bonded to the canines and first premolars, and the segment was retained by the distraction device for 6 months to allow callus ossification and stabilization of the tooth-bearing segment. Orthodontic treatment was continued to finalize the occlusion and preparing the interdental distance between the mandibular canines and the premolars for a later implant placement. The bone anchors were surgically removed after six months. Two dental implants (NobelActive NP 3.5 X 11.5 mm, Nobel Biocare, Goteborg, Sweden) were inserted without additional bone grafting. A fixed retainer was bonded between the first mandibular premolar and canine. A removable retention was applied to the maxilla. The total treatment period was 20 months (Fig. **8**).

## 3 DISCUSSION

This case report presents a 57-year-old female with a skeletal Angle Class I relation and a severe dental Class II malocclusion, which was treated successfully with AMSDO. Distraction osteogenesis is mainly used in orthopedic surgery and oral and maxillofacial surgery to repair skeletal deformities and in reconstructive surgery. Distraction osteogenesis is a process of growing new bone by mechanical stretching of the reparative bone tissue and soft tissue through incremental lengthening by a distraction device. AMSDO enables a greater range of segmental movement compared to conventional anterior segmental subapical osteotomy and allows skeletal correction of patients with a skeletal Angle Class I relation and a severe dental Class II malocclusion without compromising the facial aesthetic.

The skeletal and dental stability after AMSDO using a tooth-borne distraction device has been assessed in a long-term study disclosing a mean relapse of 8.3% at point B and 29.0% at incision inferior after a mean follow-up of 5.5 years [3]. There were no correlation between the amount of advancement and relapse [3]. However, a rotational rather than a translational advancement of the tooth-bearing alveolar segment was observed in these long-term studies, which could lead to an unfavourable inclination of the lower incisors and canines after the distraction phase [3].

A tooth-borne distraction device is commonly used for AMSDO [3, 5, 6, 8, 9, 12]. Dentoalveolar changes have been assessed after AMSDO using a tooth-borne distraction device disclosing a mean dental tipping rate of 24%, with 76% skeletal movement [10]. Hence, a bone-borne distraction device has been recommended to minimize the risk of dental tipping during the distraction phase [11]. A previously published study evaluated the amount of skeletal movement and dental tipping after AMSDO using a bone-borne distraction device revealing a mean dental tipping rate of 2.4%, with 97.6% skeletal movement [11]. Consequently, the use of a bone-borne distraction device seems to minimize the amount of dental tipping during the distraction phase. An anterior segmental subapical osteotomy with instant fixation in the desired position and a distraction procedure for the alveolar segment has been suggested as an alternative surgical method to control the inclination of the lower incisors and canines [10]. In the present case, the osteotomized tooth bearing segment was moved forward with a tooth-borne distraction device in combination with skeletal fixated bone anchors displaying limited dental tipping with satisfying skeletal movement.

The perimeter of the dental arch has been estimated after AMSDO using a bone-borne distraction disclosing a mean enlargement of the apical base of 7.9 mm and 12.7 mm of the dentoalveolar arch [11]. In the present case, the perimeter of the dental arch was adequately enlarged to facilitate placement of an implant in the created interdental distance between the canines and premolars.

The long-term soft tissue changes after AMSDO using a tooth-borne distraction device disclosed that the net effect of the soft tissue at point B is 88% of the total skeletal advancement at point B and the lower lip followed the advancement of incision inferior to 24% after 5.5 years [4]. The authors concluded that the physiological process of aging and loss of soft tissue elasticity should be considered as a reason for the soft tissue changes over time [4, 6].

The most common complications associated with AMSDO involve periodontal impairment, neurosensory disturbances of the inferior alveolar nerve and tooth injuries. Gingival recessions or root fenestrations of the lower incisors after AMSDO have never previously been reported. In contrast, a slight improvement of gingival recessions has been reported in one study [9]. However, periodontal impairment adjacent to the vertical osteotomy line has been reported in two studies [10, 11]. Minor gingival recessions of 1 mm were reported in almost half of the included patients [11], and gingival recession was observed around the teeth adjacent to the vertical osteotomy line in one-third of patients [10]. Temporary postoperative neurosensory disturbances of the oral mucosa have been reported after AMSDO [9]. However, no statistically significant differences in neurosensory status were reported between patients treated with AMSDO compared to a control group after five years [7]. Tooth injury during the vertical osteotomy procedure has been reported in one study [9]. The tooth was extracted and later replaced with an implant [9]. Thus, preoperative orthodontic root spreading is compulsory to minimize the risk of tooth injury and periodontal impairment during the vertical osteotomies.

Patient compliance and vector control are important aspects to be considered when planning AMSDO. Moreover, completely mobilization of the tooth-bearing segment is mandatory to prevent bony interferences.

## CONCLUSION

Treatment of a 57-year-old female with a skeletal Angle Class I relation and a severe dental Class II malocclusion using AMSDO has been presented and the current knowledge about this treatment modality has been discussed. Thus, the establishment of a harmonious relationship between the maxillary and mandibular arch in patients with a skeletal Angle Class I relation and a severe dental Class II malocclusion using AMSDO seems to be a predictable and applicable surgical method for selected cases and General Dental Practitioners, orthodontics and maxillofacial surgeons must have knowledge of this treatment modality.
